# Fatty acid metabolism predicts prognosis and NK cell immunosurveillance of acute myeloid leukemia patients

**DOI:** 10.3389/fonc.2022.1018154

**Published:** 2022-10-20

**Authors:** Zhitao Ye, Yishan Li, Xiaobin Tian, Yan Wei, Yanhui Yu, Kaibin Lai, Keyue Yang, Zixuan Qiu, Jianqing Lin, Meng Zhao, Dongjun Lin, Xiaojun Xu

**Affiliations:** ^1^ Department of Hematology, The Seventh Affiliated Hospital, Zhongshan School of Medicine, Sun Yat-sen University, Shenzhen, China; ^2^ Key Laboratory of Stem Cells and Tissue Engineering (Ministry of Education), Zhongshan School of Medicine, Sun Yat-sen University, Guangzhou, China; ^3^ Department of Hematology, Heping Hospital Affiliated to Changzhi Medical College, Changzhi, China; ^4^ Changzhi Medical College, Changzhi, China

**Keywords:** acute myeloid leukemia, fatty acid metabolism, prognostic model, tumor microenvironment, NK cells

## Abstract

**Background:**

Cell metabolic reprogramming is a hallmark of tumor prognosis, and fatty acid metabolism (FAM) plays a crucial role in the tumor microenvironment (TME). However, the relationship between FAM, TME, and prognosis of acute myeloid leukemia (AML) patients remains elusive.

**Methods:**

We extracted the single-cell RNA sequencing (scRNA-Seq) and bulk transcriptome data of AML patients from the TCGA and GEO databases and assessed the relationship between FAM, TME, and AML patient prognosis. We also performed functional enrichment (FE) assay to evaluate the significance of FAM in anti-AML immunosurveillance.

**Results:**

Our scRNA-Seq analysis revealed that the leukemic stem cell (LSC)-enriched population exhibited elevated levels of FAM-related genes. Using these FAM-related genes, we developed a prognostic model that accurately estimated AML patient outcome. FE analysis showed that FAM was strongly related to alterations of TME-based immunosurveillance in AML patients. More importantly, we demonstrated that FAM inhibition *via* pharmaceutical targeting of PLA2G4A, a highly expressed FAM gene in AML patients with poor prognosis, enhanced the NK cell-mediated immunosurveillance in leukemia cells.

**Conclusions:**

Leukemic stem cell (LSC)-enriched population exhibited elevated levels of FAM-related genes. We have successfully established the FAM formula that predicts AML patient prognosis and alterations in the TME-based immunosurveillance. We also found that PLA2G4A was a highly expressed FAM gene in AML patients with poor prognoses. Pharmaceutical targeting of PLA2G4A increased the expression of NKG2DL in leukemia cells *in vitro* and thus enhanced the NK cell-mediated immunosurveillance.

## Introduction

Acute myeloid leukemia (AML) is a widespread hematopoietic malignancy characterized by uncontrolled clonal expansion of primitive myeloid precursors (1). The global AML incidence has progressively increased over the years, with 75% of AML patients initially diagnosed after 60-year-old age (2). Although a diverse range of targeted therapy strategies has emerged in recent years, intensive chemotherapy remains the standard treatment for AML patients. Despite increasing complete remission (CR) rates, AML prognosis remains poor due to the high relapse rate and drug resistance (3).

Leukemic stem cell (LSC) is functionally defined by the ability to initiate and establish diseases upon transplantation (4). Similar to hematopoietic stem cells, LSCs stand at the top of the leukemia lineage by self-renewing and differentiating into proliferative leukemic cells (5). In addition, the quiescent LSCs are resistant to chemotherapeutic interventions, thus leading to their survival and disease re-establishment (6). Therefore, targeting the chemoresistance and self-renewal mechanism to eliminate LSCs is crucial for effective AML treatment.

Over time, research revealed that fatty acid metabolism (FAM) plays an essential role in AML cell survival and chemoresistance (7). Leukemic cells prefer to metabolize fatty acids to meet the augmented bioenergetic demands as fatty acid oxidation (FAO) generates more than twice as much ATP as glucose oxidation. LSCs have relatively low levels of prolyl hydroxylase 3 (PHD3), a crucial enzyme in glucose oxidation (8). Meanwhile, LSCs highly express the fatty acid transporter CD36 and the fatty acid-binding protein 4 (FABP4) that promotes fatty acid uptake and transport to fuel fatty acid lipolysis in bone marrow adipocytes (9–12), Given this evidence, targeting LSC metabolic vulnerabilities like FAM dependency may be a possible approach for eradicating chemoresistant LSCs and improving AML prognosis (13). However, the prognostic value of FAM-related genes and their relationship to the tumor microenvironment (TME) in AML are rarely reported and require further investigations (11).

Here, we identified that AML LSCs have high levels of FAM-related genes. We constructed a FAM prognostic model for better predicting AML patient outcomes and alterations in the TME-based immunosurveillance. More importantly, pharmaceutically targeting the FAM gene PLA2G4A enhanced the NK cell-mediated immunosurveillance by increasing NKG2D ligand expression in leukemia cells.

## Materials and methods

### Data acquisition and identification of the FAM-related genes

We gathered RNA sequencing data and corresponding clinical data from 553 AML patients on the GEO website (GSE37642, https://www.ncbi.nlm.nih.gov/geo/) and utilized them as the training cohort (TC) for analysis (**Table S1**). Similarly, the Cancer Genome Atlas (TCGA) dataset (n=140) with clinical information from the UCSC Xena (http://xena.ucsc.edu/) was used as the validation cohort (VC) (**Table S2**). FAM-related genes were obtained from the “c2.cp.kegg.v7.0.symbols”. Genes were selected for further investigation only if they were listed in both the TC and VC. The genes involved in the FAM formula construction are presented in **Table S3**.

### scRNA-Seq data processing

Raw data, with accession number GSM5400788, was downloaded from the Gene Expression Omnibus (GEO) websiteViable primary human AML cells (CD33+/CD45+/AnnexinV-) from mice bone marrow of PDX mice before chemotherapy, or accepted chemotherapy treatment were included in the analysis. The chemotherapy treatment consists in 5 days of AraC treatment (30 mg/kg for PDXs) by intra-peritoneal injection, or with 7 days of venetoclax treatment (100 mg/kg) by oral gavage We conducted normalization, dimensionality reduction, and clustering using the Seurat 3.2.3 R package. Cell filtration was done such that the system identified > 500 and < 5,000 genes and < 5% of total UMIs mapped to the mitochondrial genome. The data was normalized by dividing the UMI counts per gene into the total UMI counts in the corresponding cells, followed by log-transformation to achieve results, then scaling and centering. We next performed dimensionality reduction on the cells using Stochastic Neighbor Embedding (t-SNE). Pseudotime trajectory was then assessed *via* monocle2, depending on the Seurat clustering. Next, we retrieved the signature genes from each cluster with the Seurat function FindMarkers with the “wilcox” test. The Gene Ontology (GO) and plots were then employed *via* cluster Profiler and the ggplot2 R package. Lastly, gene summaries were pre-stratified by DEG fold change values *via* Seurat, and the gene sets were acquired *via* the GO database.

### Construction and validation of the FAM formula

To establish an effective prognostic prediction model, we used the univariate Cox regression analysis of 553 GEO-LAML patients in the TC to construct the FAM formula. The clinical characteristics of these patients are presented in **Table S1**. The FAM formula was generated with data from the multivariate analysis with the lowest Akaike Information Criterion (AIC) value. Lastly, we computed the risk score of individual patients using the FAM formula as follows: Risk Score = e ^ sum (normalized individual FAM-associated gene levels multiplied by the corresponding regression coefficiency). The same FAM formula was used to compute the risk scores of the training cohort (TC) and validation cohort (VC) patients. Next, both TC and VC patients were stratified into a high risk (HR) or low risk (LR) cohort, based on the same threshold value. Subsequently, the Kaplan-Meier analysis was used to assess overall survival (OS) between the HR and LR cohorts in VC. We next generated the predictive nomogram A hybrid using the “rms” R package that integrated both the FAM formula and corresponding clinical patient profile to estimate the AML patient OS at the 1-, 2-, and 3-year time points. Lastly, the calibration curve and consistency index (C-index) were employed to evaluate the predictive ability of the generated nomogram.

### Gene set and functional enrichment analysis

GSEA v4.1.0 (http://software.broadinstitute.org/gsea/login.jsp) was employed to determine relevant physiological networks between the HR and LR cohorts, as evidenced by the FAM formula and c5.go.bp.v7.5.symbols gene sets. The GSEA analysis was performed in both the TC and VC. A nominal p-value < 0.05 was set as the significance threshold. The GO analysis was conducted to explore biological processes, while the Kyoto Encyclopedia of Genes and Genomes (KEGG) network analysis was conducted to determine signaling pathways.

### Analysis of immune cells involved in leukemia

The XCELL algorithm was utilized to quantify various types of tumor-invading immune cells in AML patients from the TC and VC cohorts, and a p-value < 0.05 was considered significant. Next, we evaluated each category of immune cells to evaluate the differential tumor microenvironment (TME) profiles between the HR and LR cohorts.

### mRNA isolation and qPCR

Total mRNA was extracted with the MagZolTM Reagent (R4801-03, Magen) following kit directions, and the transcript purity and quantification were evaluated *via* NanoDrop (Thermo Scientific) prior to qPCR. To conduct RT-qPCR, transcript samples were converted to cDNA with the TransScript All-in-One First-Strand cDNA Synthesis SuperMix (AT341, Transgen), and qPCR was carried out with the SYBR Green I Master Mix reagent (11203ES03, YEASEN) in the Bio-Rad CFX96 TouchTM Real-Time PCR Detection system. The primers used for the qPCR are listed in **Table S4**. The expression levels of the NKG2DL were normalized using GADPH as the internal control.

### Apoptosis analysis

The three leukemia cell lines, including THP1 (CTCC-001-0044, Meisen), U937 (CTCC-001-0027, Meisen) and HL60 (CTCC-001-0025, Meisen), were grown in RPMI 1640 containing 10% FBS. After treatment with AACOCF3(GC16115, GLPBIO) at 25μM for 48 hours, we evaluated cellular apoptosis with Annexin-V (640907, Biolegend) by flow cytometric analysis.

### *In vitro* NK killing assay

NK92(CTCC-001-0016, Meisen) cell lines were grown in RPMI 1640 containing 10% FBS, 10% horse serum (Solarbio), and 100 ng/ml IL-2 (Peprotech).Firstly, we labelled the leukemia cells with CFSE for 30 minutes and washed off CFSE carefully. Then we treated AML cell lines with 5μM AACOCF3(GC16115, GLPBIO) for 48 hours, after which we washed off the remaining AACOCF3 and continued culturing in the presence or absence of NK92 cells for 60 hours before counting live leukemia cells by flow cytometric analysis. The killing efficiency of NK92 cells were calculated as follows: Killing efficiency= (the number of leukemia cells grown without NK92 cells - number of leukemia cells grown with the indicated frequency of NK92 cells)/number of leukemia cells grown without NK92 cells.

### Statistical analyses

The Chi-squared test was used to evaluate the correlation between the FAM formula and the corresponding patient clinical profile. R (Version 4.1.0) and SPSS (Version 23.0) were employed for data analyses. Data from cell lines *in vitro* were compared with the Student’s t tests, and p < 0.05 was set as the significance threshold.

## Results

### FAM-related genes are highly expressed in chemoresistant LSCs

**Figure 1:**
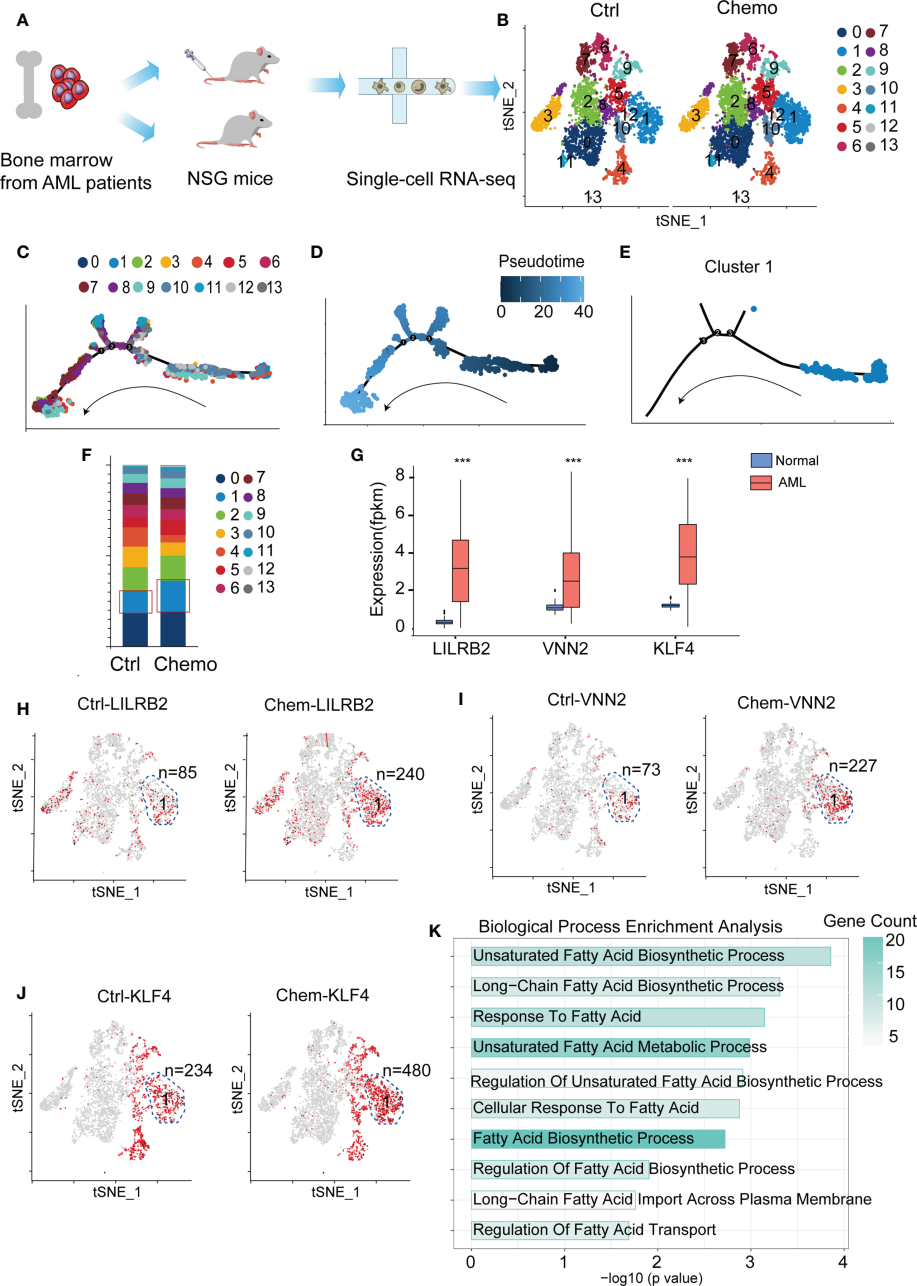
Fatty acid metabolism (FAM)-related genes are highly enriched in chemoresistant leukemic stem cells (LSCs). **(A)** A flow chart depicting the process of scRNA-Seq data acquisition. **(B)** Cluster the untreated (n=2 mice) and chemotherapy-treated leukemia cells (n=4 mice) with the t-SNE dimensionality reduction analysis. **(C–E)** The pseudotime trajectory analysis of the cells in the chemotherapy group. **(F)** Cell quantification of each cluster before and after chemotherapy. **(G)** Bar plot depicting expressions of KLF4, VNN2, and LILRB2 in AML patients and normal people. The p-value was calculated by unpaired two-tailed Student t-tests, p < 0.05, p < 0.01, p < 0.001. **(H–J)** LSC markers (LILRB2, VNN2 and KLF4) are enriched in the cells of cluster 1. The LILRB-, VNN2-, and KLF4-high cells are highlighted in red, and the n number depicts the red cell number in cluster 1 before and after chemotherapy. **(K)** GO enrichment of the upregulated genes in cluster 1. Data are displayed as specific values or mean ± SD.

AML patient-derived xenografted mice were treated with or without chemotherapy, and leukemia cells were collected and proceeded for scRNA-Seq (**Figure 1A**). We used these scRNASeq data deposited in the GEO website for t-SNE dimensionality reduction analysis and revealed 14 clusters based on their gene profile (**Figure 1B**). We next used the pseudotime ordering analysis to construct the cell lineage differentiation trajectory. Compared with other clusters, cluster 1 is located at the root of the trajectory (**Figures 1C–E** and **Supplementary Figure 1A**), and the cell number of cluster 1 is increased after chemotherapy (**Figure 1F**), suggesting that cluster 1 is the leukemic stem cell (LSC)-enriched population. We further checked whether LSC markers were enriched in cluster 1 before and after chemotherapy. We chose LILRB2 (14), VNN2 (15), and KLF4 (16) as LSC markers since they exhibited higher expression levels in AML patients compared with normal volunteers in TCGA database and the Genotype-Tissue Expression (GTEx) project (**Figure 1G**). The number of LILRB2-high, VNN2-high and KLF-high cells in cluster 1 are 85, 73, and 234 respectively before chemotherapy and increased to 240, 227, and 480 after chemotherapy (**Figures 1H–J**). These results confirm that cluster 1 is the LSC population related to AML relapse. Subsequently, we chose the upregulated genes in cluster 1 to perform GO analysis and revealed that fatty acid metabolism (FAM) is highly enriched (**Figure 1K**). In summary, by analyzing the scRNA-Seq data we identified that cluster 1 is the AML LSC population and has upregulated FAM-related genes.

### The FAM formula accurately predicts AML patient prognosis

**Figure 2:**
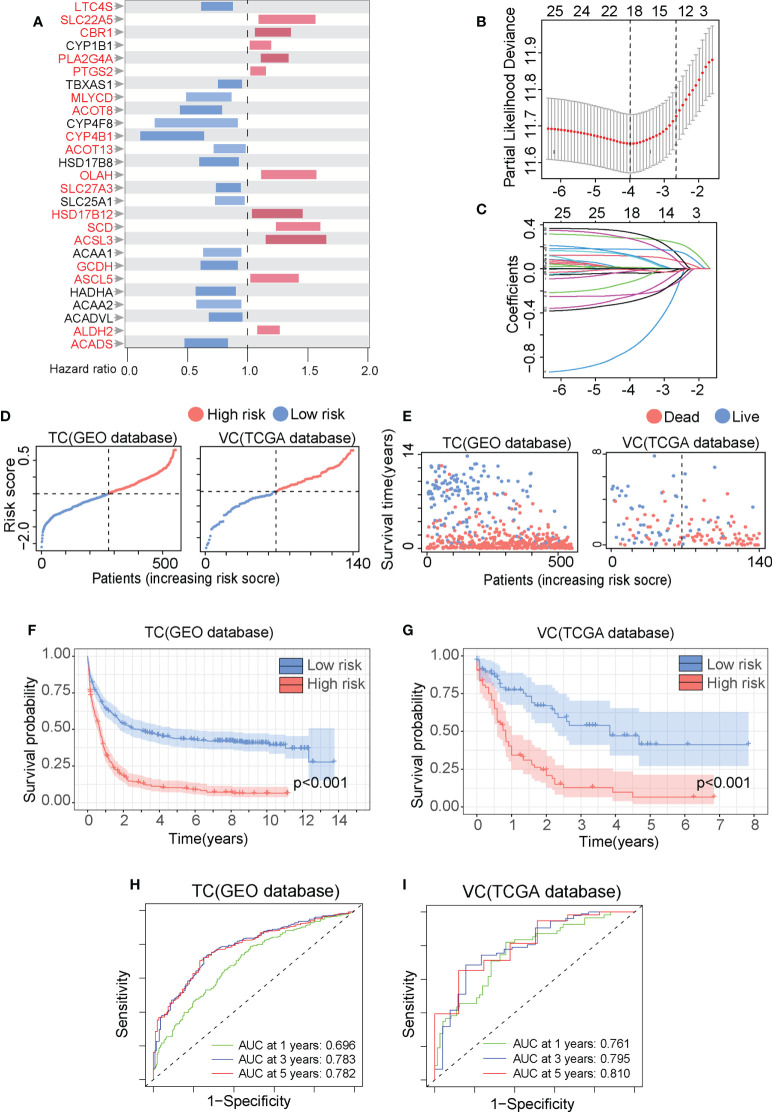
The fatty acid metabolism (FAM) formula accurately predicts AML patient prognosis. **(A)** The overall survival (OS) of 553AML patients in the GEO database was analyzed by the univariate Cox regression with the 201 FAM-related genes and summarized in Forest plots. **(B)** The FAM-related genes were analyzed by the least absolute shrinkage and selection operator (LASSO) regression model based on the minimal criteria. **(C)** The FAM-related gene in the LASSO regression analysis was calculated for coefficient. **(D, E)** The metabolic risk score distribution **(D)** and the survival outcome (SO) analysis I of the training cohorts (TC) and validation cohorts (VC). **(F, G)** The Kaplan-Meier survival curves of the HR and LR patients in the TC and VC. **(H, I)** The time-dependent ROC analyses of the FAM prognostic model to estimate the 1-, 3-, and 5-year OS of TC and VC patients.

We established a prognostic prediction model termed the FAM formula to elucidate the relationship between FAM and AML patient prognosis. The clinical features of our training cohorts (TC, GEO database, N=553) and validation cohorts (VC, TCGA database, N=140) are summarized in **Tables S1**, **S2**. First, we employed 201 FAM-related genes from the c2.all.v7.0.symbols (**Table S3**) to perform the univariate Cox regression analysis with the TC database and identified 27 genes with significant prognostic values (**Figure 2A**). Next, these genes were further analyzed with the LASSO Cox algorithm to construct a prognostic model (**Figures 2B, C**), which reduced the significant gene number to 18 (highlighted in **Figure 2A**). The risk score was calculated as follows: risk score = ACADS levels*(-0.0385781574616528) + ALDH2 levels*(0.115943870930126) + ACSL5 levels*(0.116279878492424) + GCDH levels*(-0.023543074746657) + ACSL3 levels*(0.0034823102467818) + SCD levels*(0.278308214066157) + HSD17B12 levels*(0.125378923880509) + SLC27A3 levels*(-0.247889819557295) + OLAH levels*0.329315900149358 + ACOT13 levels*(-0.119336558434181) + CYP4B1 levels*(-0.730901151964483) + ACOT8 levels*(-0.233925370148131) + MLYCD levels*(-0.29877179590025) + PTGS2 levels*(0.0130085245854858) + PLA2G4A levels*(0.177305593342932) + CBR1 levels*(0.273311180808465) + SLC22A5 levels*(0.00204092301483134) + LTC4S levels*(-0.00758524180721233).

We set the median value of the TC risk score calculated by the FAM formula as the threshold to distinguish between high risk (HR) and low risk (LR) cohorts. Data in **Figures 2D, E** illustrates the risk score distribution and survival status of the HR and LR cohorts in the TC and VC database. We Use the Kaplan-Meier analysis to demonstrate that the HR cohort experienced considerably worse overall survival (OS) than the LR cohort (**Figures 2F, G**, p-value<0.001). We validate the FAM formula by drawing ROC curve in which the area under ROC curve (AUC) is positively related with the prognosis accuracy. The AUC for the 1-year, 3-year, and 5-year survival rates in the TC were 0.696, 0.783, and 0.782, respectively (**Figure 2H**). The AUC for the 1-year, 3-year, and 5-year survival rates in the VC were 0.761, 0.795, and 0.810, respectively (**Figure 2I**). The AUC values in both the TC and VC are greater than 0.65, indicating that the FAM formula was highly effective in predicting AML patient prognosis.

### Integrating clinicopathological characteristics into the FAM formula optimizes its predictive ability of AML patient prognosis

**Figure 3:**
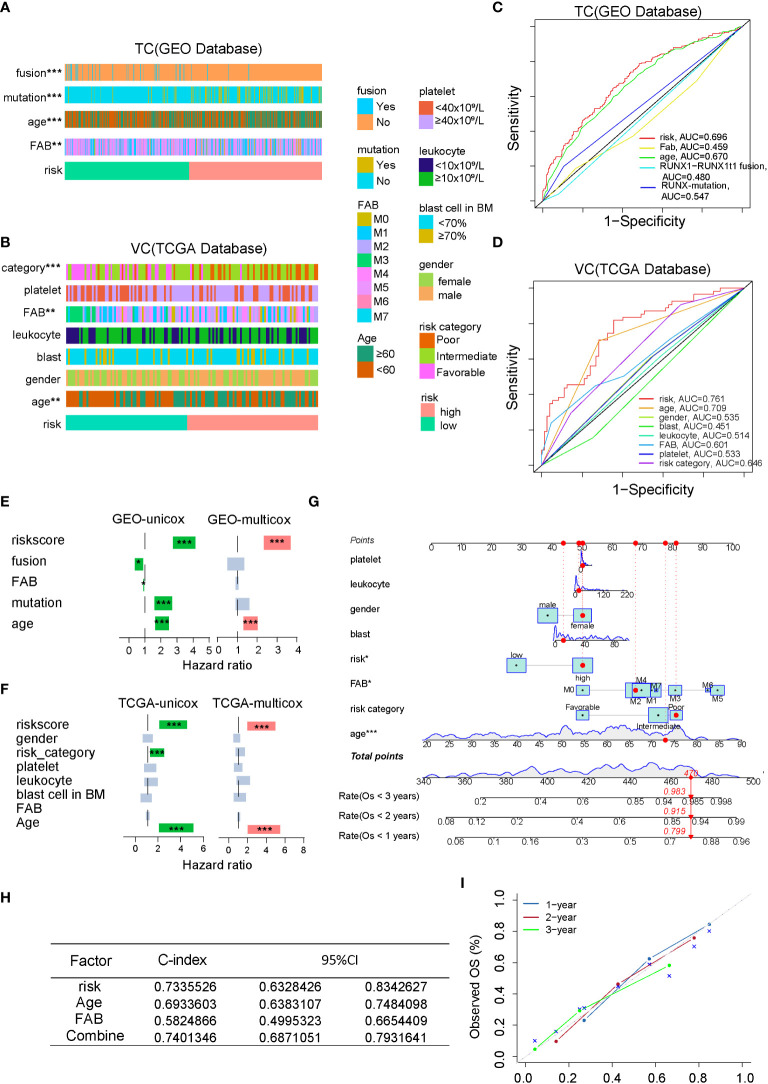
Combining the clinicopathological characteristics with the FAM formula optimizes the predictive capacity of the nomogram. **(A, B)** A strap plot summarized various clinicopathological features and FAM-related risk score, and the correlation between clinical features and the FAM risk score was analyzed by chisq.test. *p < 0.05,**p < 0.01, ***p < 0.001. **(C, D)** Evaluation of the prognostic prediction accuracy *via* the area under the time-dependent receiver operating characteristic (ROC) curve (AUC). **(E, F)** Stand-alone prognostic ability of the FAM formula or clinical features in the TC and VC. **(G)** Nomogram plot, based on the risk score and other clinicopathological patient profiles in the VC. **(H)** The consistency index (C-index) of the nomogram. **(I)** The calibration curve of the nomogram.

To optimize the predictive ability of the FAM formula, we combined the FAM formula with other clinicopathological characteristics to construct a prognostic nomogram. First, we compared the relationship between the FAM formula and the clinicopathological characteristics listed in **Tables S1**, **S2**, such as runx1 fusion protein, runx1 mutation, FAB subtype, age, platelet count, leukocyte count, blast cell count, and gender. As shown in **Figures 3A, B**, AML patients with younger age and M3 FAB subtype are more likely to have a FAM-based low risk score. To compare the sensitivity and specificity of the FAM formula with other clinicopathological characteristics in the prognostic model, we conducted ROC analysis and calculated the AUCs. The FAM formula risk scores were the highest in both the TC and VC, suggesting that FAM is most accurate for predicting AML prognosis (**Figures 3C, D**). We next used univariate and multivariate Cox regression analyses to test the independence of these signatures (**Figures 3E, F**). Based on our univariate analysis, the 18-gene FAM formula was strongly correlated with AML patient prognosis. In particular, in the TC database hazard ratio (HR) = 3.389, 95% confidence interval (CI) = 2.733−4.202, P< 0.001,and in the VC database HR = 3.117, 95% CI = 2.121−4.580. This 18-gene signature was determined to be an independent stand-alone risk factor by multivariate analysis for AML patient outcome in the TC: HR = 2.879, 95% CI = 2.291−3.617, P< 0.001, and the VC: HR = 3.142, 95% CI = 1.983−4.982. To optimize the clinical value and application probability of the FAM formula in predicting AML patient prognosis, we utilized the “rms” package of the R software to combine the clinicopathological characteristics with the risk score to generate a new signature named Combine. We generate a nomogram to compare these signatures for estimating the 1-, 2-, and 3-year prognosis of AML patients (**Figure 3G**). The consistency index (C-index) and the calibration curve of the nomogram were performed to evaluate the predictive efficiency and accuracy of the nomogram. In the Combine group, the C-index = 0.74 and represents the highest among all groups (**Figure 3H**), indicating that it has the best predictive ability. The calibration curve showed that the curves of 1-, 2-, and 3-year are very close to the diagonal dotted line, confirming that the Combine signature has a high predictive ability in the nomogram (**Figure 3I**). In summary, the Combine signature that incorporates the FAM formula and clinicopathological profiles of patients has excellent stability and accuracy in predicting AML prognosis, suggesting that it has the potential for application in clinics.

### The FAM formula predicts alterations in the tumor microenvironment (TME)

**Figure 4:**
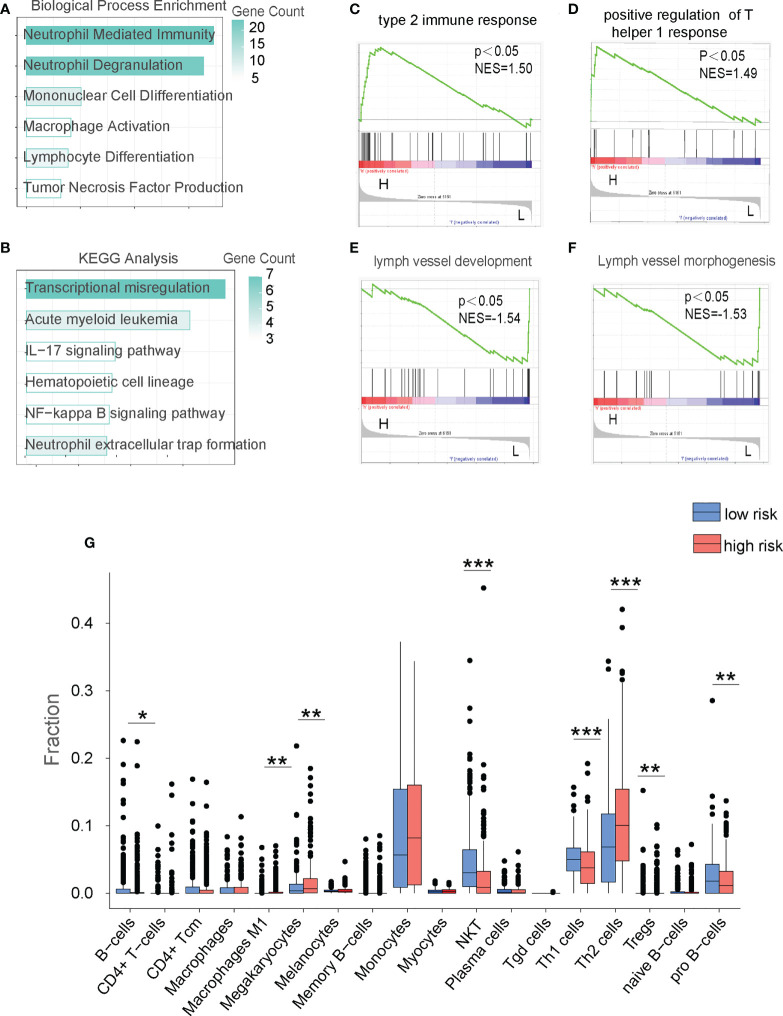
The FAM formula identifies alterations in the immune microenvironment of AML patients in the TC database. **(A)** GO analysis of the low risk (LR) and high risk (HR) cohorts in the TC. **(B)** KEGG network analysis of the LR and HR cohorts in the TC. **(C–F)** GSEA analysis of LR and HR cohorts in the TC. **(G)** Analyze the immune cell populations of the LR and HR patients using the XCELL algorithm in the TC. *p < 0.05,**p < 0.01, ***p < 0.001.

To determine the underlying mechanism behind the opposite prognoses in the HR and LR cohorts, we performed the functional enrichment (FE) analysis. We first compared the HR and LR cohorts to identify the differentially expressed genes (DEGs) with the p-value cut off < 0.05. We then conducted Gene Ontology (GO), Kyoto Encyclopedia of Genes and Genomes (KEGG), and Gene Set Enrichment Assay (GSEA) to establish underlying enriched processes. Based on our GO analysis, DEGs between LR and HR patients in the TC and VC databases were primarily enriched in immune-related processes (**Figure 4A**; **Supplementary Figure 2**). In addition, KEGG analysis revealed that immune-regulatory pathways, including IL-17 signaling and NF-κB signaling, were highly enriched in the HR cohort (**Figure 4B**). And GSEA analysis revealed that the immune-related biological processes were highly enriched in the HR cohort (**Figures 4C–F**; **Supplementary Figures 2C–F**). As tumor microenvironment (TME) plays an important role in the development, proliferation, and survival of leukemia blasts (17), we next analyzed TME of AML patients. Based on the signaling patterns of the HR and LR patients, the XCELL algorithms (18) can delineate various immune cell populations located in the TME. As shown in **Figure 4G** and **Supplementary Figure 2G**, both the VC and TC demonstrated alteration in immune cell composition, with an evident NKT cell decrease in the HR cohort. Collectively, TME is altered in HR cohorts, which may be a critical factor in determining AML patient prognosis and providing a chance to foster novel AML treatment strategies.

### PLA2G4A inhibition enhances the killing efficiency of NK cells against LSCs

**Figure 5:**
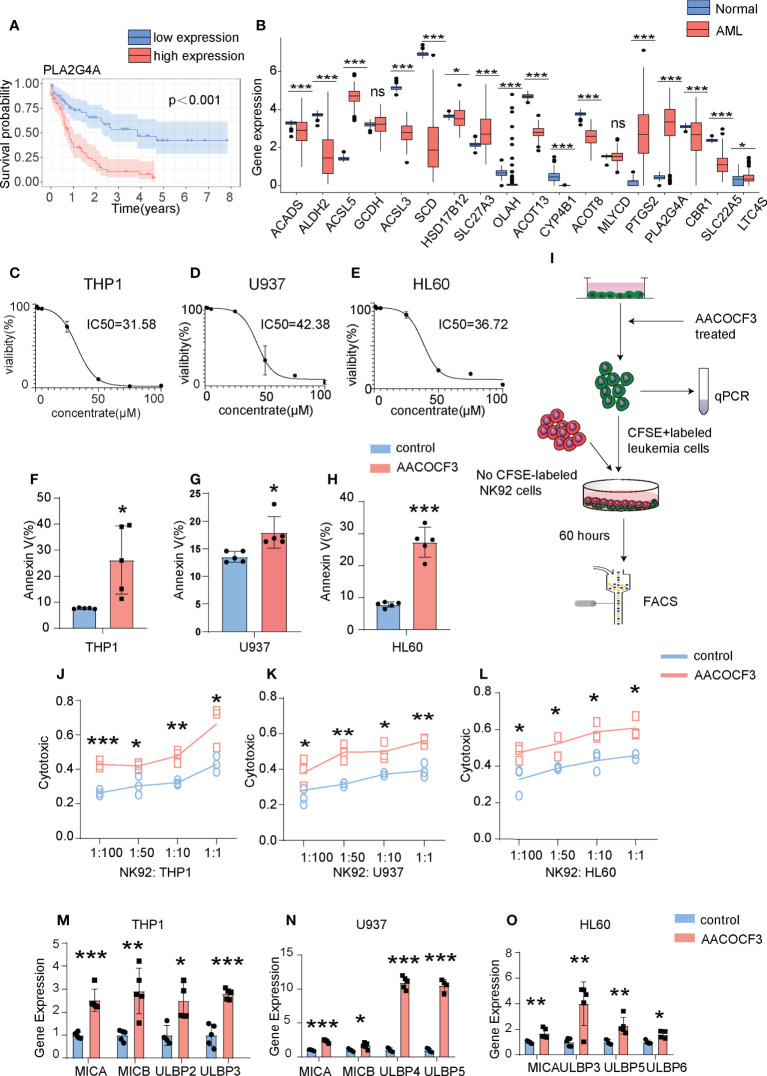
PLA2G4A inhibition enhances NK cell-mediated cytotoxicity against AML cells. **(A)** Kaplan-Meier analysis of patients in the VC based on PLA2G4A expression. **(B)** The Barplot illustrates the expression levels of individual FAM formula genes between leukemia and normal groups from the TCGA database. **(C–E)** Three leukemia cell lines (THP1, U937 and HL60) were treated with increasing dosages of PLA2G4A inhibitor AACOCF3 for recording the cell viability (n=3 independent experiments). **(F–H)** Apoptotic analysis of THP1, U937 or HL60 cells (n=5 independent experiments). **(I)** A flow chart depicting leukemia cell lines co-cultured with NK92 cells, followed by FACS analysis. **(J–L)** THP1, U937, or HL60 cells was treated with or without AACOCF3 before co-culturing with NK92 cells, and leukemic cell cytotoxicity was calculated (n=3 independent experiments). **(M–O)** Realtime PCR results show the expression of NKG2DL genes in leukemia cell lines treated with the PLA2G4A inhibitor AACOCF3 (n=3 independent experiments). All experiments were analyzed using unpaired two-tailed Student’s t-tests. *P<0.05, **P<0.01, ***P<0.001. ns, no significance.

Based on the aforementioned FE analysis, we speculated that alteration in the immune microenvironment might be a critical factor in determining AML prognosis. To assess whether FAM regulates AML immune microenvironment, we performed overall survival (OS) analysis with the single gene of the FAM formula and found that 7 out of 18 genes have significant predictive value on AML patient prognosis with a p-value cut-off <0.05 in VC (TCGA database). Among them, enrichment of PLA2G4A most strongly represented the poor prognosis (**Figures 5A, B**, **S3**). Then, we compared the expression of eighteen FAM formula genes between AML patients and normal individuals. PLA2G4A, a member of the cytosolic phospholipase that catalyzes the hydrolysis of membrane phospholipids to release arachidonic acid, increased by 7.21 fold, representing one of the most upregulated genes in AML patients with poor prognosis (**Figures 2A**, **5A**). These results indicate that PLA2G4A may be a crucial FAM enzyme involved in AML progress. Thus, we treated leukemia cell lines THP1, U937, and HL60 with PLA2G4A inhibitor AACOCF3, an analog of arachidonic acid that inhibits the PLA2G4A phospholipase activity by competing for the active catalytic site. AACOCF3 is the trifluoromethyl ketone derivative of arachidonic acid. This compound is a selective inhibitor of soluble PLA2 and Ca2+ independent PLA2 in human cells. (19, 20). The IC50 values of THP1, U937, and HL60 are 31.58μM, 42.38μM, and 36.72μM, respectively, indicating that AACOCF3 had a low cytotoxic effect on leukemia cells at low dosages but induced cell death at high dosages (**Figures 5C–E**). In parallel, Annexin-V+ apoptotic cell number was highly increased in THP1, U937, and HL60 cells when exposed to the high concentration of AACOCF3 (25μM) (**Figures 5F–H**), suggesting that high dose AACOCF3 directly induced leukemia cells death. As we identified an evident decrease of NKT cells in the high-risk TME, we examined the effect of PLA2G4A inhibition on NK-mediated cytotoxicity against leukemia cells. We labeled THP1, U937, and HL60 cells with CFSE and treated them with low dose AACOCF3 (5μM), in which leukemia cells should still be viable according to the survival curve in **Figures 5C–E**. We then co-cultured AACOCF3 pretreated leukemia cells with NK cell line NK92 before conducting FACS and quantitative PCR analyses (**Figure 5I**). AACOCF3 treatment significantly enhanced the cytotoxicity of NK92 against leukemia cells (**Figures 5J–L**). The NK group 2D (NKG2D) is a cell surface receptor to activate the NK-mediated cytotoxic effect when binding to NKG2D ligands such as MICA, MICB, and ULBP family members. We examined the expression of NKG2D ligands in leukemia cell lines treated with low concentration AACOCF3 (5μM) and found that expression levels of the MICA, MICB, and ULBP family were all significantly elevated (**Figures 5M–O**). Overall, our results revealed that inhibiting phospholipase PLA2G4A enhances NK-mediated immunosurveillance toward leukemia cells.

## Discussion

Our findings demonstrated that the leukemic stem cell (LSC)-enriched population exhibited elevated levels of FAM genes. Using these FAM genes, we constructed a prognostic model that accurately evaluates AML patient prognosis. We also observed that FAM correlates with immunosurveillance alteration in AML patients. Pharmaceutically inhibiting the FAM enzyme PLA2G4A increased expressions of NKG2D ligands in leukemia cells, thus enhancing NK cell-mediated cytotoxicity against leukemia cells.

Although studies on metabolic reprogramming during AML progression are gradually increasing (21), the effect of metabolic drugs on life expectancy is generally limited. Fatty acid metabolism (FAM) is a well-recognized hallmark of AML prognosis (22), but the relationship and the underlying molecular mechanisms between FAM-related genes and AML prognosis are not elucidated. Herein, we analyzed the scRNA-Seq data of patient-derived xenografted AML cells and identified that the LSC-enriched population has elevated expression of FAM-related genes. Based on the hypothesis that FAM is required for AML progression, we constructed a prognostic model named the FAM formula composed of eighteen FAM-related genes to predict AML patient prognosis. We have performed the univariate cox regression analysis and found that some FAM genes like ACADS, GCDH, SLC27A3, ACOT13, LTC4S were considered the protective factor with favorable prognosis. In contrast, other FAM genes like ALDH2, ASCL5, ASCL3, SCD, HSD17B12, OLAH, CYP4B1, ACOT8, MLYCD, PTGS2, PLA2G4A, CBR1 and SLC22A5 were considered as risk genes with poor prognosis. Among these genes, high expressions of ALDH2, SCD, and PLA2G4A were experimentally confirmed as the risk genes with poor prognosis *in vitro* or *in vivo* (20). ALDH2, the aldehyde dehydrogenase in the mitochondria of leukemia cells that suppresses formaldehyde accumulation, was closely related to AML relapse (23). SCD is the enzyme that converts saturated fatty acids to monounsaturated fatty acids (24). Inhibiting the activity of SCD induces leukemia cell apoptosis and may be a novel way to eradicate leukemia stem cells. Interestingly, PLA2G4A is a biomarker predicting the poor prognosis of AML patients (20), which is in line with our findings (**Figure 5A**). Furthermore, we compared the clinicopathological characteristics with FAM-predicted risk factors and found that the poor prognosis factors, such as runx1 mutation and old age, are highly enriched in the high-risk (HR) cohort (**Figures 3A, B**), confirming that FAM indeed plays a role in AML progression. We further integrated the FAM formula with clinical characteristics to construct the Combine formula that more accurately predicts AML prognosis and might have the potential for clinical application in the future.

More importantly, our mechanistic study identified that FAM in LSCs influences AML progression by suppressing the immune microenvironment. LSCs require an immunosuppressive microenvironment for survival during chemotherapy and disease re-establishment (25). Stromal cells like MSCs secrete TGFβ to reduce NKG2D expression and inactivate NK cells and other T cell subpopulations (26). Moreover, LSCs themselves downregulate NKG2D ligand expressions to escape NK-mediated immunosurveillance (27, 28). Our results in **Figure 4B** and **Supplementary Figure 2G** indicate that the HR AML patients identified by the FAM formula exhibited markedly reduced NKT cells and other immune cell populations in both the training cohort (TC) and validation cohort (VC). These results suggest that immune microenvironment alteration correlates with AML patient prognosis. More importantly, we found that FAM suppression by pharmaceutically targeting PLA2G4A enhanced NK cell immunosurveillance towards AML cells *in vitro*. Mechanistically, PLA2G4A inhibition upregulated NKG2D ligand expressions for boosting NK-mediated anti-leukemic cytotoxicity. Taken together, our study revealed that FAM suppression might be a novel strategy for optimizing AML treatment by enhancing immune surveillance.

## Data availability statement

The original contributions presented in the study are included in the article and **Supplementary Material**. Further inquiries can be directed to the corresponding authors.

## Ethics statement

Both TCGA database, GEO database, and Gtex database belong to public databases and the patients involved in these databases have obtained ethical approval. There are no ethical issues and other conflicts of interest occur in our study.

## Author contributions

ZY performed most of the experiments. XT, YW, and KL collected the data from the database and wrote the manuscript. YY and YL analyzed the data. KY, ZQ, and JL cultured the cells. MZ, DL, and XX designed the present study, analyzed the data and revised the manuscript. All authors contributed to the article and approved the submitted version.

## Funding

This work was supported by Sanming Project of Medicine in Shenzhen(No.SZSM201911004), Shenzhen Fundamental Research Program(No.JCYJ20180307150408596), Scientific and Technological Innovation Programs of Higher Education Institutions in Shanxi (2021L353), the Natural Science Foundation for Young Scientists of Shanxi Province (20210302124089).

## Acknowledgments

We acknowledge TCGA, GEO and Gtex database for providing their platform and contributors for uploading their meaningful datasets.

## Conflict of interest

The authors declare that the research was conducted in the absence of any commercial or financial relationships that could be construed as a potential conflict of interest.

## Publisher’s note

All claims expressed in this article are solely those of the authors and do not necessarily represent those of their affiliated organizations, or those of the publisher, the editors and the reviewers. Any product that may be evaluated in this article, or claim that may be made by its manufacturer, is not guaranteed or endorsed by the publisher.

## Supplementary material

Supplementary Figure 1 Pseudotime trajectory analysis of the cells in the chemotherapy group. The pseudotime trajectory analysis of each cluster in the chemotherapy group.

Supplementary Figure 2 The FAM formula identifies alterations in the immune microenvironment of AML patients in VC database. **(A)** GO analysis of the low risk (LR) and high risk (HR) cohorts in the VC. **(B)** KEGG network analysis of the LR and HR cohorts in the VC. **(C–F)** GSEA analysis of LR and HR cohorts in the VC. **(G)** Analyze the immune cell populations of the LR and HR patients in the VC using the XCELL algorithm.

Supplementary Figure 3 Kaplan-Meier analysis of AML patients based on the expression level of individual FAM-related genes. Kaplan-Meier analysis of AML patients in the VC based on the expression levels of SCD, SLC22A5, ACADS, MLYCD, ACSL3, and CBR1. Significance: *P<0.05, **P<0.01, ***P<0.001.
