# Supplementary material for: Fatty acid metabolism predicts prognosis and NK cell immunosurveillance of acute myeloid leukemia patients
Source: Front Oncol. 2022 Oct 20;12:1018154. doi: 10.3389/fonc.2022.1018154 (PMC9633260; doi:10.3389/fonc.2022.1018154)
Supplement: Supplementary file 1 [file Table_1.docx]

|  | high | low | p |
| --- | --- | --- | --- |
| n | 276 | 277 |  |
| Age (%) |  |  |  |
| ≥60 | 157 (56.884) | 102 (36.823) | <0.001 |
| ＜60 | 119 (43.116) | 175 (63.177) |  |
| Fab (%) |  |  | NA |
| M0 | 19 (6.884) | 3 (1.083) |  |
| M1 | 61 (22.101) | 52 (18.773) |  |
| M2 | 79 (28.623) | 85 (30.686) |  |
| M3 | 0 (0.000) | 26 (9.386) |  |
| M4 | 52 (18.841) | 69 (24.910) |  |
| M5 | 39 (14.130) | 27 (9.747) |  |
| M6 | 11 (3.986) | 11 (3.971) |  |
| M7 | 3 (1.087) | 0 (0.000) |  |
| unknow | 12 (4.348) | 4 (1.444) |  |
| RUNX1- RUNX1t1 fusion (%) |  |  |  |
| true | 4 (1.449) | 26 (9.386) | <0.001 |
| false | 272(98.551) | 251 (90.614) |  |
| RUNX1-mutation (%) |  |  | <0.001 |
| true | 62 (22.464) | 13 (4.693) |  |
| false | 195 (70.652) | 224 (80.866) |  |
| unknow | 19 (6.884) | 40 (14.440) |  |

**Table S1． Clinical characteristics of patients in the training cohort**
