# Supplementary material for: Fatty acid metabolism predicts prognosis and NK cell immunosurveillance of acute myeloid leukemia patients
Source: Front Oncol. 2022 Oct 20;12:1018154. doi: 10.3389/fonc.2022.1018154 (PMC9633260; doi:10.3389/fonc.2022.1018154)
Supplement: Supplementary file 2 [file Table_2.docx]

|  | high | low | p |
| --- | --- | --- | --- |
| n | 73 | 67 |  |
| Age (%) |  |  | 0.007 |
| ≥60 | 42 (57.534) | 23 (34.328) |  |
| ＜60 | 31 (42.466) | 44 (65.672) |  |
| Gender |  |  | 0.126 |
| male | 45 (61.644) | 32 (47.761) |  |
| female | 28 (38.356) | 35 (52.239) |  |
| Leukocyte (%) |  |  | 0.662 |
| ＜10 x10^9/L | 27 (36.986) | 27 (40.299) |  |
| ≥10x10^9/L | 46 (63.014) | 39 (58.209) |  |
| unknow | 0 (0.000) | 1 (1.493) |  |
| FAB (%) |  |  | NA |
| M0 | 10 (13.699) | 4 (5.970) |  |
| M1 | 15 (20.548) | 15 (22.388) |  |
| M2 | 17 (23.288) | 17 (25.373) |  |
| M3 | 0 (0.000) | 15 (22.388) |  |
| M4 | 15 (20.548) | 13 (19.403) |  |
| M5 | 12 (16.438) | 3 (4.478) |  |
| M6 | 2 (2.740) | 0 (0.000) |  |
| M7 | 1 (1.370) | 0 (0.000) |  |
| unknow | 1 (1.370) | 0 (0.000) |  |
| Platelet (%) |  |  | 0.172 |
| ≥40 x10^9/L | 46 (63.014) | 34 (50.746) |  |
| ＜40 x10^9/L | 27(36.986) | 33 (49.254) |  |
| Blast cell in BM |  |  | 0.681 |
| ≥70% | 17 (23.288) | 13 (19.403) |  |
| ＜70% | 56 (76.712) | 54 (80.597) |  |
| Risk category |  |  | ＜0.001 |
| favorable  intermediate  poor  unknow | 3 (4.110)  43 (58.904)  26 (35.616)  1 ( 1.4) | 28 (41.791)  33 (49.254)  5 (7.463)  1 ( 1.5) |  |

**Table S2． Clinical characteristics of patients in the validation cohort**
