# Supplementary material for: Fatty acid metabolism predicts prognosis and NK cell immunosurveillance of acute myeloid leukemia patients
Source: Front Oncol. 2022 Oct 20;12:1018154. doi: 10.3389/fonc.2022.1018154 (PMC9633260; doi:10.3389/fonc.2022.1018154)
Supplement: Supplementary file 3 [file Table_3.docx]

**Table S3． Fatty acid metabolism related genes involved in the construction of prognostic model**

| CPT1A | CPT1C | ACADS | ALDH1B1 | ACADSB | ACADL | ALDH2 |
| --- | --- | --- | --- | --- | --- | --- |
| CYP4A11 | ACAT2 | ACAT1 | ACAA2 | HADH | HADHB | HADHA |
| ADH7 | ADH6 | ACSL6 | ADH1B | ADH1C | ECHS1 | ADH5 |
| ALDH3A2 | ACSL5 | ADH1A | EHHADH | ALDH7A1 | ACOX3 | ACSL1 |
| CPT2 | CPT1B | ACOX1 | ECI2 | ECI1 | ACSL3 | ACSL4 |
| BAAT | ELOVL5 | ELOVL6 | ACOT2 | YOD1 | ELOVL2 | FADS1 |
| PECR | SCD5 | SCD | ACADM | CYP4A22 | ALDH9A1 | ACAA1 |
| TECR | FADS2 | HACD1 | ACOT1 | ACOT7 | HSD17B12 | HACD2 |
| ELOVL1 | HACD3 | SLC25A1 | ACSBG1 | ELOVL4 | ELOVL3 | ACSBG2 |
| HSD17B3 | PPT1 | ACLY | MORC2 | SLC27A3 | CBR4 | OLAH |
| ELOVL7 | FASN | HACD4 | HSD17B8 | TECRL | PPT2 | HTD2 |
| ACACA | NDUFAB1 | ACSM3 | DECR1 | ACAD10 | ACOT13 | PCCB |
| MECR | ACOT9 | MCEE | PCTP | MMUT | MMAA | DBI |
| THEM4 | ACOT11 | ACSF2 | ACAD11 | CYP2J2 | CYP4B1 | CYP4F2 |
| ACOT12 | ACSM6 | PCCA | ACBD7 | THEM5 | ACBD6 | CYP4F12 |
| CYP4F8 | CYP4F3 | CYP2F1 | CYP2A7 | ACOT8 | CD36 | PLCB2 |
| ABCD1 | HSD17B4 | ACOT4 | ACOT6 | DECR2 | GNA11 | FFAR1 |
| GNA14 | GNAQ | PLCB1 | GNA15 | FFAR2 | FFAR3 | FFAR4 |
| SLC27A6 | SLC27A1 | LCN9 | LCN1 | SLC27A4 | LCN15 | LCN12 |
| ACACB | APOD | CROT | ALOX5 | DPEP1 | TBXAS1 | PTGS2 |
| CRAT | GGT1 | SLC25A17 | ABCC1 | ALOX12 | PTGES3 | LTA4H |
| PON3 | PON2 | PRKAG2 | PTGR1 | PTGDS | PHYH | ACBD5 |
| PPARD | SCP2 | PLA2G4A | HAO2 | FAAH | EPHX2 | PTGIS |
| HACL1 | ALOX5AP | CYP1B1 | CYP2C9 | CYP2C8 | PTGR2 | SLC27A2 |
| CYP1A1 | CYP1A2 | AC092724.1 | DPEP3 | PTGES2 | PTGES | THRSP |
| ACOXL | CYP2U1 | PRXL2B | CBR1 | PRKAA2 | MAPKAPK2 | HPGDS |
| RXRA | HPGD | MID1IP1 | FAAH2 | CYP2C19 | DPEP2 | GPX4 |
| ACOX2 | GPX2 | SLC25A20 | ALOXE3 | ALOX12B | CYP8B1 | ACBD4 |
| SLC22A5 | AWAT1 | LTC4S | GPX1 | AMACR |  |  |
