# Supplementary material for: Fatty acid metabolism predicts prognosis and NK cell immunosurveillance of acute myeloid leukemia patients
Source: Front Oncol. 2022 Oct 20;12:1018154. doi: 10.3389/fonc.2022.1018154 (PMC9633260; doi:10.3389/fonc.2022.1018154)
Supplement: Supplementary file 4 [file Table_4.docx]

| **Primers** | **Primer sequence (5’-3’)** |  |
| --- | --- | --- |
| **Gene** | **Forward Primer** | **Reverse Primer** |
| MICA | AGGGTTTCTTGCTGAGGTACA | GGTCTCTCTGTCCCATGTCTTA |
| MICB | TCTTCGTTACAACCTCATGGTG | TCCCAGGTCTTAGCTCCCAG |
| ULBP1 | TAAGTCCAGACCTGAACCACA | TCCACCACGTCTCTTAGTGTT |
| UPBP2 | AGCAACTGCGTGACATTCAG | GCCATCCTATACAGTCTCCCA |
| UPBP3 | TCTATGGGTCACCTAGAAGAGC | TCCACTGGGTGTGAAATCCTC |
| UPBP4 | GCACTTGGGGAGAATTGACCC | ACATCTCGACTTGCAGAGTGG |
| UPBP5 | GACAGCTACCAAATAGCGAAGC | GGTAAGGAGTGTGAGTCGTCT |
| UPBP6 | TCATCCCTAAGTTCAGACCTGG | GGACTGACGGGTGTGACTG |
| GAPDH | GGAGCGAGATCCCTCCAAAAT | GGCTGTTGTCATACTTCTCATGG |

**Table S4． The primer sequences used in this study.**
