# Supplementary figures and images for: Fatty acid metabolism predicts prognosis and NK cell immunosurveillance of acute myeloid leukemia patients
Source: Front Oncol. 2022 Oct 20;12:1018154. doi: 10.3389/fonc.2022.1018154 (PMC9633260; doi:10.3389/fonc.2022.1018154)

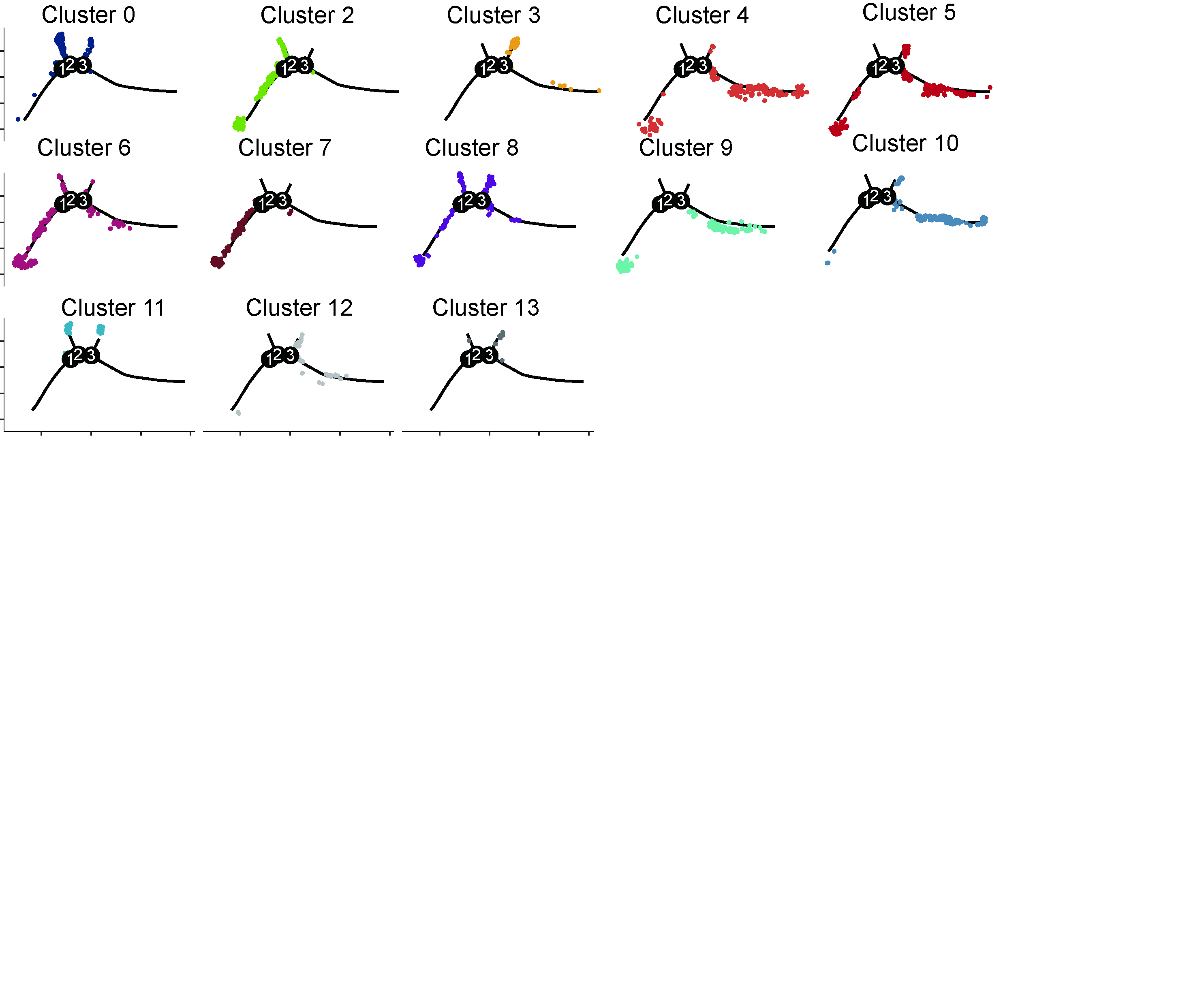

Supplement: Supplementary Figure 1 — Pseudotime trajectory analysis of the cells in the chemotherapy group. The pseudotime trajectory analysis of each cluster in the chemotherapy group. [file Image_1.tif]

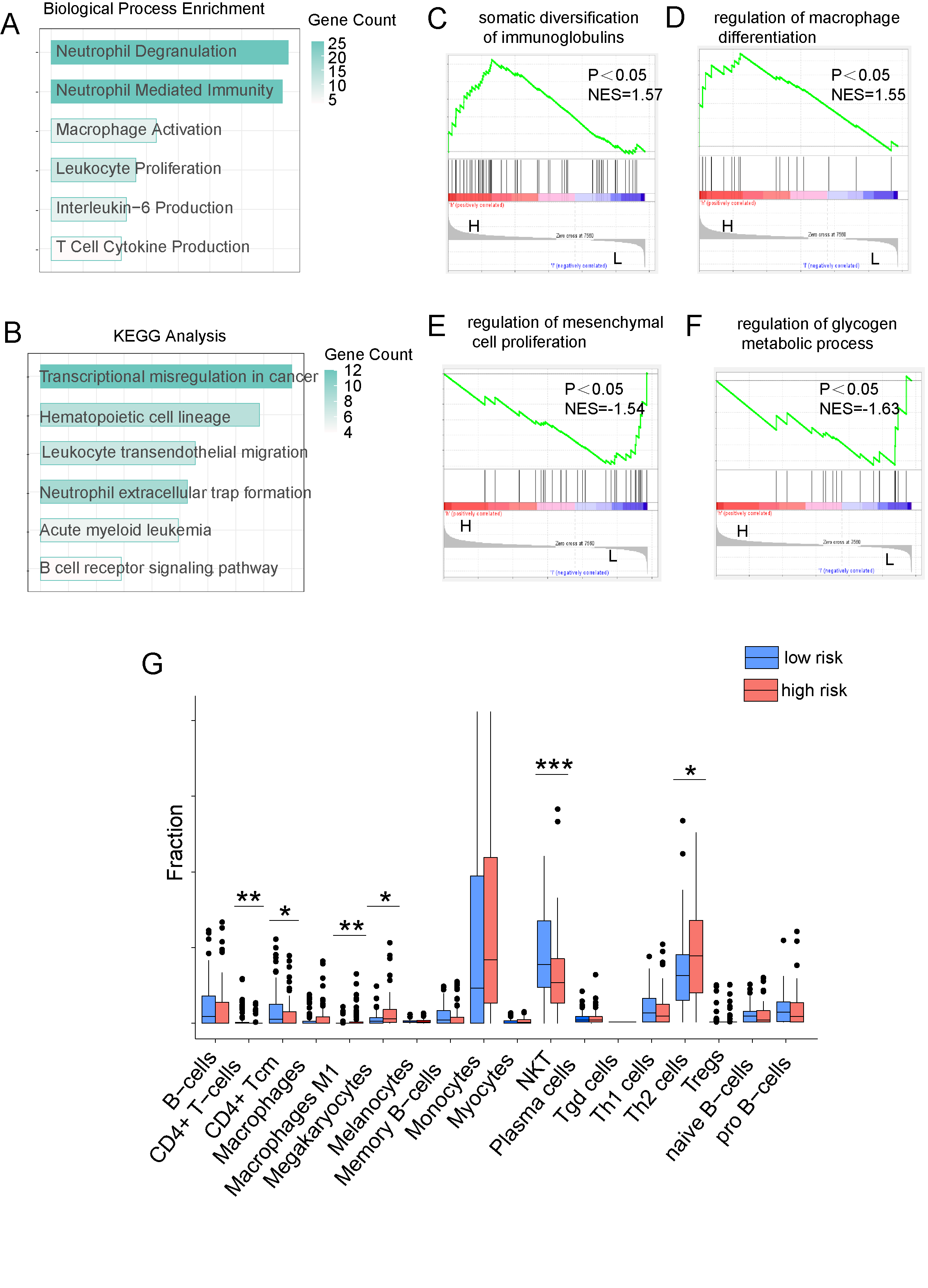

Supplement: Supplementary Figure 2 — The FAM formula identifies alterations in the immune microenvironment of AML patients in VC database. (A) GO analysis of the low risk (LR) and high risk (HR) cohorts in the VC. (B) KEGG network analysis of the LR and HR cohorts in the VC. (C–F) GSEA analysis of LR and HR cohorts in the VC. (G) Analyze the immune cell populations of the LR and HR patients in the VC using the XCELL algorithm. [file Image_2.tif]

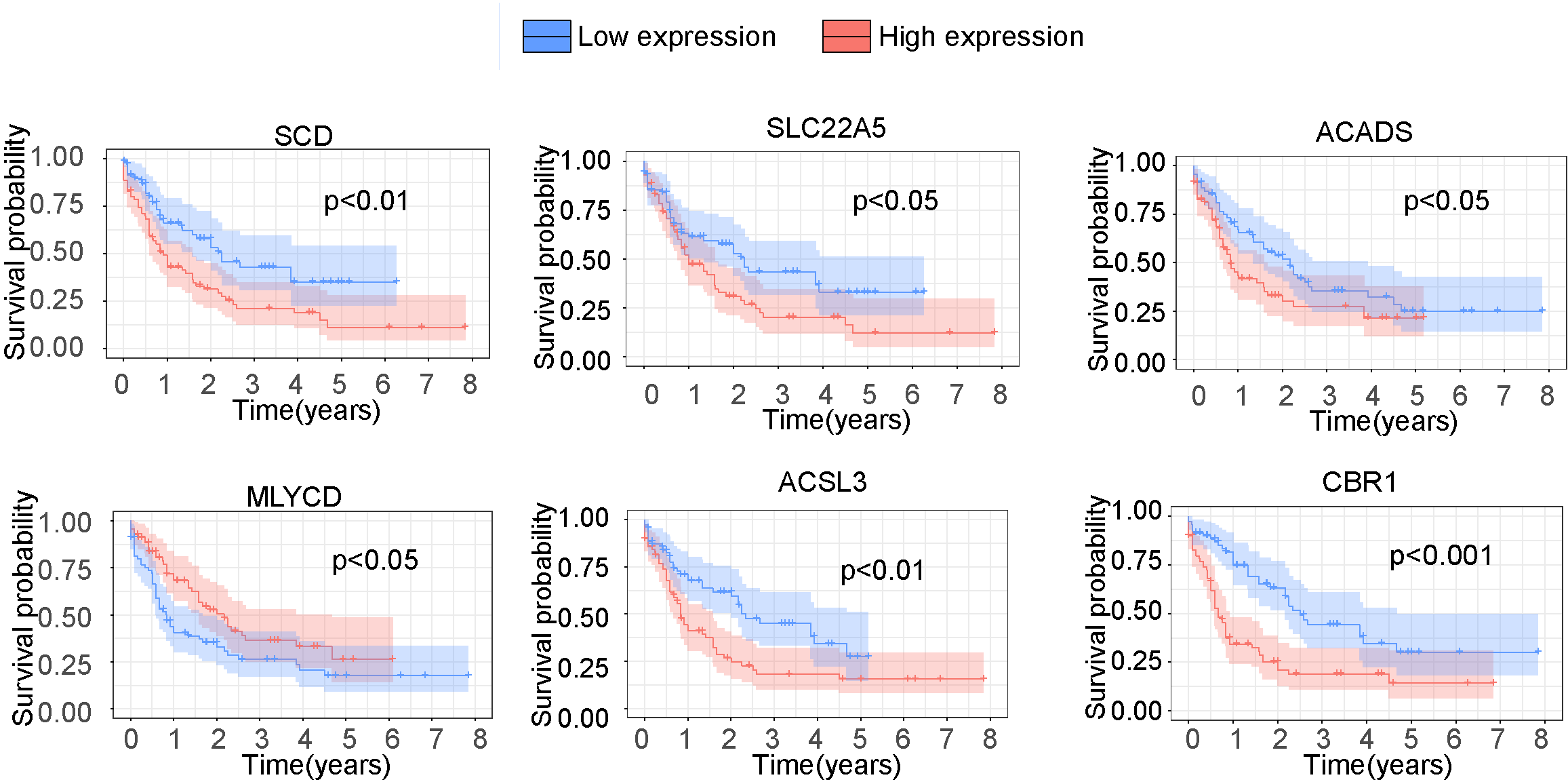

Supplement: Supplementary Figure 3 — Kaplan-Meier analysis of AML patients based on the expression level of individual FAM-related genes. Kaplan-Meier analysis of AML patients in the VC based on the expression levels of SCD, SLC22A5, ACADS, MLYCD, ACSL3, and CBR1. Significance: *P<0.05, **P<0.01, ***P<0.001. [file Image_3.tif]
